# Need for BCG Vaccination to Prevent TB in High-Incidence Countries and Populations

**DOI:** 10.3201/eid2603.191232

**Published:** 2020-03

**Authors:** Shalini Pooransingh, Sateesh Sakhamuri

**Affiliations:** The University of the West Indies, St. Augustine, Trinidad and Tobago

**Keywords:** tuberculosis, vaccination, BCG, tuberculosis and other mycobacteria, World Health Organization, bacteria, vaccines, bacille Calmette-Guérin

## Abstract

An estimated one quarter of persons worldwide are infected with *Mycobacterium tuberculosis*. In 2018, the World Health Organization issued revised guidance on BCG vaccine for high-risk groups. The World Health Organization should consider guiding countries on a case-by-case basis in developing appropriate BCG policies to deliver equitable healthcare and protect public health.

In 1993, the World Health Organization (WHO) recognized tuberculosis (TB) as a global emergency (*1*). Twenty-five years later, TB remains a major public health challenge. It is the single leading infectious cause of death globally. An estimated one quarter of the world’s population is infected with *Mycobacterium tuberculosis* (*2*). In 2017, 10 million persons became ill with TB, and 1.6 million died of it. That year, an estimated 1 million children became ill with TB, and 230,000 died (*3*).

Ending the TB epidemic by 2030 is a primary goal of WHO and, if achieved, will contribute to WHO’s Sustainable Development Goal 3, which is concerned with health (*4*). In keeping with Pillar 1 of the End TB Strategy, no opportunity to control TB should be missed. The treatment for latent infection in combination with treatment measures for active disease or with preexposure vaccination can substantially decrease TB incidence (*5*).

Until a new TB vaccine is developed, *M. bovis* bacille Calmette-Guérin (BCG) remains the only effective vaccine for TB (*6*,*7*). BCG’s overall efficacy, including cost-effectiveness, has been questioned by studies that were confounded by the cross-reactivity of antigens and the absence of measures to exclude latent infection before vaccination. None of these studies considered BCG’s primary preventive effect on drug-resistant TB and tangential benefits, such as avoidance of prolonged treatment and unwanted adverse effects. BCG’s importance is again increasing in light of new, encouraging evidence about its efficacy and because of the limited availability of alternative new anti-TB strategies.

BCG effectiveness in preventing the life-threatening forms of TB in children is unquestionable. Vaccination at birth or shortly thereafter protects against disseminated and pulmonary TB in young children (*4*). Vaccination is cost-effective in the following groups: infants in settings where TB incidence rates are >20 cases/100,000 population or 5 cases/100,000 smear-positive cases per year; school-aged children in high-risk settings in school-based catch-up programs; and settings with low TB incidence where vaccination of specific populations, such as immigrants from high-incidence countries and healthcare workers, is selectively administered. High global coverage and widespread use of BCG in routine infant vaccination programs could prevent >115,000 TB deaths per birth cohort during the first 15 years of life (*8*).

WHO therefore recommends that, in countries with a high incidence of TB, a single dose of BCG should be provided to all infants at or soon after birth as part of the national schedule. In countries with low TB incidence, BCG may be limited to neonates and infants in recognized high-risk groups or to older children who are skin test–negative for TB infection.

Despite clear evidence and WHO recommendations, however, global BCG administration practices appear to be arbitrary (*9*). Among 180 countries, 154 (86%) reported universal BCG vaccination. Of the other 26 countries, 20 reported having had a national BCG policy for everyone in the past, and the remaining 6 countries reported having selective vaccination for persons in high-risk groups. Among the 30 high TB–incidence countries globally, BCG coverage ranged from 53% to 99%; coverage was <80% in 6 high-incidence countries (*3*).

Governments committed to achieving universal health coverage should revise their national BCG vaccination policies to reflect newly available evidence. If health is genuinely considered a human right, this right might be realized only if governments provide the necessary services for equitable healthcare for all sectors of society. Equitable healthcare would mean BCG vaccine for all in high-incidence countries and selective vaccination for high-risk populations in low-incidence countries.

WHO is unfailing in its technical support to countries through activities such as developing guidelines, standards, and checklists, as well as supporting national and regional workshops. However, what might be needed is for regional and country WHO offices to perhaps be more proactive and more prescriptive in assisting with the adaptation of global strategies to the local country situation and with their follow-up. Such action by WHO regional and country offices may assist countries with weak health information and surveillance systems and limited human resource technical capacity, both of which might limit the timely adaptation of national policies to international best practice guidance and to rapidly changing country demographics.

The WHO Multisectoral Accountability Framework to Accelerate Progress to End Tuberculosis (*10*) is timely; it supports accountability of stakeholders at all levels (global, regional, and country). However, critical indicators concerning screening and vaccination appear to be missing.

Neglecting End TB Strategy initiatives should no longer be accepted. Countries should not abandon BCG entirely. WHO should take an active role in guiding countries on a case-by-case basis in developing appropriate BCG policies to deliver equitable healthcare, thereby protecting public health and achieving the goals of the End TB Strategy.
